# Knowledge discovery of drug data on the example of adverse reaction prediction

**DOI:** 10.1186/1471-2105-15-S6-S7

**Published:** 2014-05-16

**Authors:** Pinar Yildirim, Ljiljana Majnarić, Ozgur Ilyas Ekmekci, Andreas Holzinger

**Affiliations:** 1 Department of Computer Engineering, Faculty of Engineering & Architecture, Okan University, Istanbul, Turkey; 2 School of Medicine, University J.J. Strossmayer Osijek, Crotia; 3 Institute for Medical Informatics, Statistics & Documentation, Medical University of Graz, Graz, Austria

**Keywords:** Knowledge Discovery, Data Mining, adverse reactions and allergy (ARA), k-means algorithm

## Abstract

**Background:**

Antibiotics are the widely prescribed drugs for children and most likely to be related with adverse reactions. Record on adverse reactions and allergies from antibiotics considerably affect the prescription choices. We consider this a biomedical decision-making problem and explore hidden knowledge in survey results on data extracted from a big data pool of health records of children, from the Health Center of Osijek, Eastern Croatia.

**Results:**

We applied and evaluated a k-means algorithm to the dataset to generate some clusters which have similar features. Our results highlight that some type of antibiotics form different clusters, which insight is most helpful for the clinician to support better decision-making.

**Conclusions:**

Medical professionals can investigate the clusters which our study revealed, thus gaining useful knowledge and insight into this data for their clinical studies.

## Background

Antibiotics are the drugs most widely prescribed to children and are most likely to be associated with allergic and adverse reactions [1-4]. A reaction to a drug is known an allergic reaction if it involves an immunologic reaction to a drug. It may happen in the form of immediate or non-immediate (delayed) hypersensitivity reactions. Immediate reactions are usually mediated with IgE antibodies (often elevated in persons with inherited susceptibility to allergic diseases, called atopy), whereas non-immediate reactions can be mediated with several other immune mechanisms [5]. The clinical manifestations of antibiotic allergy include skin reactions (varying from local and mild general to severe general reactions), organ-specific reactions (most commonly occurring in the form of blood dyscrasias, hepatitis and interstitial nephritis) and systemic reactions (usually corresponding with anaphylaxis) [5]. Many reactions to drugs mimic symptoms and signs of the allergic reactions, although being caused with non-immunologic mechanisms. In many cases, also, pathologic mechanisms remain completely unclear. This is the reason why these reactions are often considered together and commonly named adverse reactions and allergy (ARA) [6]. This term is especially appropriate for use in primary health care setting, where patients who had experienced ARA on antibiotics have rarely been referred to testing. Moreover, diagnostic tests have some limitations and are only standardized for penicillin allergy [6].

Antibiotic classes with higher historical use have been shown to have higher allergy prevalence [7]. Published papers on frequency, risk factors and preventability of this medical problem in the general population, and especially in children, are scarce. Available data implicate female sex, frequent use, older age, insufficient prescribing strategy and monitoring of prescribed medications, as the primary factors accounting for higher prevalence of ARA on antibiotics among adults. Similar data for children are completely absent [8].

The aim of this study is to explore hidden knowledge in the survey data extracted from health records on adverse reactions and allergy on antibiotics in children in the town of Osijek, Eastern Croatia. We plan to obtain some serious and useful information in electronic health records that are not easily recognized by researchers, clinicians and pharmaceutical companies.

### Related work

There have been many works carried out for knowledge discovery on diseases and drug adverse events associations. Kadoyama et al. searched the FDA's AERS (Adverse Event Reporting System) and performed a study to reveal whether the database could offer the hypersensitivity reactions caused by anticancer agents, paclitaxel, docetaxel, procarbazine, asparaginase, teniposide and etoposide. They used some data mining algorithms, such as "proportional reporting ratio (PRR), the reporting odds ratio (ROR) and the empirical Bayes geometric mean (EBGM) to identify drug-associated adverse events and consequently, they found some associations" [9].

Tsymbal et al. investigated antibiotics resistance data and proposed a new ensemble machine learning technique, "where a set of models are built over different time periods and the best model is selected"[10]. They analyzed the data collected from the Burdenko Institute of Neurosurgery in Russia and the dataset consisted of some features such as: patient and hospitalization related information, pathogen and pathogen groups and antibiotics and antibiotic groups. Their experiments with the data show "that dynamic integration of classifiers built over small time intervals can be more effective than" the best single learning algorithm applied "in combination with feature selection", which gives the best known accuracy for the considered problem domain [10].

Lamma et al. "described the application of data mining techniques in order to automatically discover association rules from microbiological data and obtain alarm rules for data validation"[11]. Their dataset consists of " information about the patient such as sex, age, hospital unit, the kind of material (specimen) to be analyzed (e.g., blood, urine, saliva, pus, etc.), bacterium and its antibiogram"[11]. They applied the Apriori algorithm to the dataset and developed some interesting rules [11].

Harpaz et al. reported on an approach that automatically searches whether a specific adverse event (AE) is caused by a specific drug based on the content of PubMed citations[12]. A drug-ADE classification method was initially developed to detect neutropenia based on a pre-selected set of drugs. This method was then applied to a different set of 76 drugs to determine if they caused neutropenia. For further proof of concept they applied this method to 48 drugs to determine whether they caused another AE, myocardial infarction. These results showed that AUROC was 0.93 and 0.86 respectively [12].

Lin et al. offered an interactive system platform for the detection of ADRs(Adverse Drug Reaction). By integrating an ADR data warehouse and innovative data mining techniques, the proposed system not only provides OLAP style multidimensional analysis of ADRs, but also allows the interactive discovery of relations between drugs and symptoms, known a drug-ADR association rule, which can be further developed using other factors of interest to the user, such as demographic information. The experiments indicate that interesting and valuable drug-ADR association rules can be efficiently mined [13].

Warrer et al. investigated studies that "use text-mining techniques in narrative documents stored in electronic patient records (EPRs) to investigate ADRs"[14]. They searched PubMed, Embase, Web of Science and International Pharmaceutical Abstracts without restrictions from origin until July 2011. They included empirically based studies on "text mining of electronic patient records (EPRs) that focused on detecting ADRs, excluding those that investigated adverse events not related to medicine use"[14]. They extracted information on "study populations, EPR data sources, frequencies and types of the identified ADRs, medicines associated with ADRs, text-mining algorithms used and their performance"[14]. "Seven studies, all from the United States, were eligible for inclusion in the review. Studies were published from 2001, the majority between 2009 and 2010"[14]. "Text-mining techniques varied over time from simple free text searching of outpatient visit notes and inpatient discharge summaries to more advanced techniques involving natural language processing (NLP) of inpatient discharge summaries"[14]. "Performance appeared to increase with the use of NLP, although many ADRs were still missed"[14]. "Due to differences in study design and populations, various types of ADRs were identified and thus we could not make comparisons across studies"[14]. "The review underscores the feasibility and potential of text mining to investigate narrative documents in EPRs for ADRs"[14]. However, more empirical studies are needed to evaluate whether text mining of EPRs can be used systematically to collect new information about ADRs [14].

Forster et al. identified studies evaluating electronic ADE detection from the MEDLINE and EMBASE databases[15]. They included "studies if they contained original data and involved detection of electronic triggers using information systems"[15]. "They abstracted data regarding rule characteristics including type, accuracy, and rational "[15]. Honigman et al. also developed a program that combines four computer search methods, including text searching of the electronic medical record, to detect ADEs in outpatient settings[16]. Although further refinements to their methodology should improve the overall accuracy of detection, their data demonstrate that the methodology of combining several searching tools can be successful in retrospectively detecting with moderate sensitivity ADEs in the electronic medical record [16].

The influence of resident gut microbes on xenobiotic metabolism has been explored at different levels throughout the past five decades[17]. "However, with the advance in sequencing and pyrotagging technologies, pointing out the influence of microbes on xenobiotics had to evolve from assessing direct metabolic effects on toxins and botanicals by conventional culture-based techniques to elucidating the role of community composition on drugs metabolic profiles through DNA sequence-based phylogeny and metagenomics"[17]. Following the completion of the Human Genome Project, the rapid, substantial growth of the Human Microbiome Project (HMP) opens new horizons for studying how microbiome compositional and functional variations affect drug action, fate, and toxicity (pharmacomicrobiomics), notably in the human gut. The HMP continues to characterize the microbial communities associated with the human gut, determine whether there is a common gut microbiome profile shared among healthy humans, and investigate the effect of its alterations on health. Saad et al. offered "a glimpse into the known effects of the gut microbiota on xenobiotic metabolism, with emphasis on cases where microbiome variations lead to different therapeutic outcomes"[17]. They discussed a few examples representing how the microbiome interacts with human metabolic enzymes in the liver and intestine[17]. In addition, they attempted to envisage a roadmap for the future implications of the HMP on therapeutics and personalized medicine [17].

Some researchers also investigated gene-disease associations. Arrais et al. presented a study n innovative computational method that addresses the problem of using disperse biomedical knowledge to select the best candidate gene associated with a disease[18]. The method that they offered uses a network representation of current biomedical knowledge that includes biomedical concepts such as genes, diseases, pathways and biological process [18]. Furlong also reviewed recent literature on network analysis related to disease [19].

## Methods

### The study population and data sources

The study was done on the population of 1491 children (769 children of the school age, 7-18 years old, the rest of the preschool age), all patients in the same Health Center in the town of Osijek, Eastern Croatia, cared for by a family physician and a primary pediatrician teams.

Data were extracted from the health records of these children. Knowledge of risk factors for ARA on antibiotics in children are scarce. In making a choice for data collection, a co-author physician used personal knowledge on factors influencing the immunologic reactions together with information from the studies on risk factors for allergic diseases in children [20-27]. Data extraction, from the patients health records, was guided by a multi-item chart, in an advance prepared by this co-author. In addition, parents of children recorded on ARA on antibiotics were interviewed by telephone, on a family history of ARA on antibiotics and other allergic and chronic diseases, in which pathogenesis, in a great part, immunologic mechanisms are involved. Data were summarized.

Registered information on ARA on antibiotics was found in health records of 46 children, out of a total of 1491 children screened, implicating the overall prevalence of ARA on antibiotics of 3,15%. However, higher prevalence was found in children of the school age (4,9%), then in those of the preschool age (1,1%), data probably reflecting the cumulative incidence rates with age. When the incidence data were however estimated, it has been shown that ARA on antibiotics, in our study population, can be expected to occur predominantly in preschool age (33/46 cases, 71,1%).

Of registered ARA events, almost all were mild-moderate skin reactions. Only one case was in need for hospitalization (a 18-year-old girl, treated with the combination of amoxicillin and clavulonic acid). All data, including descriptions of ARA events (upon which classification of severity reaction was made) and diagnoses of diseases, were based on the native physicians' records.

### Clustering analysis by k-means algorithm

Cluster analysis is one of the important data analysis methods in data mining research. "The process of grouping a set of physical or abstract objects into classes of similar objects is called clustering. A cluster is a collection of data objects that are similar to one another and are dissimilar to the objects in other clusters" [28]. Cluster analysis has been widely used in numerous applications, including pattern recognition, data analysis, image processing and biomedical research.

There are some distance measures used in cluster analysis. The widely used distance measure is Euclidean distance, which is defined as:

$$d\left(x,y\right)=\sqrt{\overset{\text{i}}{\underset{\text{i}=1}{\sum }}{\left(\text{Xi}-\text{Yi}\right)}^{2}}$$

Euclidean distance satisfy the following mathematic requirements of a distance function:

1. d(*x,y*) ≥ 0: Distance is a nonnegative number

2. d(*x,x*) = 0: The distance of an object to itself is 0.

3. d(*x,y*) = d(*y,x*): Distance is a symmetric function.

4. d(*x,y*) ≤ d(*x,h*) + d(*h,y*): Going directly from *object × *to object *y *in space is no more than making a detour over any other object *h*(triangular inequality)[28,29].

In this study, we use the k-means algorithm to survey results on adverse reactions and allergy (ARA) on antibiotics in children. The k-means algorithm is a type of partitioning algorithm and is simple and effective. The k-means algorithm is widely used due to easy implementation and fast execution. "Let *X *= {x_i_}, *i *= 1,...,n be the set of *n *d-dimensional points to be clustered into a set of *K *clusters, *C *= {*c*_k_, *k *= 1,...,*K*}. K-means algorithm finds a partition such that the squared error between the empirical mean of a cluster and the points in the cluster is minimized. Let *µk *be the mean of cluster *c*_k"_[28,29]. The squared error (SE) between *µ_k _*and the points in cluster *c*_k _is defined as:

$$\text{SE}=\underset{\text{xi}\in \text{ck}}{\sum }{∥\text{Xi}-\mu_{\text{k}}∥}^{2}.$$

The goal of k-means is to minimize the sum of the squared error (SSE) over all k clusters. The formula of SSE is as follows:

$$\text{SSE}=\overset{K}{\underset{k=1}{\sum }}\underset{\text{xi}\in \text{ck}}{\sum }{∥\text{Xi}-\mu_{\text{k}}∥}^{2}$$

"K-means starts with an initial partition with *k *clusters and assign patterns to clusters so as to reduce the squared error"[28,29]. Since the squared error always decrease with an increase in the number of clusters *k *(with *SE = 0 *when *k *= *n*), it can be minimized only for a fixed number of clusters.

The pseudo code of a k-means algorithm is as follows:

1. arbitrarily choose *k *objects as the initial cluster centers

2. repeat

3. (re)assign each object to the cluster to which the object is the most similar, based on the mean value of the objects in the cluster

4. update the cluster means, i.e., calculate the mean value of the objects for each cluster

5. until no change [28], [29].

## Results

We selected samples from the survey results and created a dataset. Table 1 lists the antibiotics used in the dataset. The dataset consists of 26 attributes and 42 instances (Table 2, Table 3 and Table 4). The k-means algorithm was used to explore some hidden clusters in the dataset. WEKA 3.6.8 software was used. "WEKA is a collection of machine learning algorithms for data mining tasks and is an open source software"[30,31]. The software consists of tools for data pre-processing, classification, regression, clustering, association rules and visualization [30,31].

**Table 1:** Type of antibiotics used in survey

Type of antibiotics	
**Short name used in the dataset**	**Full name and information**

ampicillin	eritrom

cef& pen	cefalosporins & penicillin

pen&klav	penicillin & amoxicillin+clavulanic acid

klav	amoxicillin+clavulanic acid - a broad-spectrum

azitrom	azithromycin - a macrolide group

cef	cefalosporins - a broad-spectrum

fenoksi	fenoksimetil penicillin - per os penicillin, a narrow-spectrum

cefuroks	cefuroxime - the second generation of cefalosporins

pen	penicillin

sulfa	sulfamethoxazole

eritrom	erythromycin - a macrolide antibiotic of an older generation

**Table 2:** The attributes used in the dataset (1-9)

No	Attribute	Description	Type
1	Age	The patient's age	Numeric

2	Age of ARA	Age when the allergic/adverse reaction on antibiotics occurred	Numeric

3	Type of antibiotic	Generic name of the antibiotic by which the allergic reaction was provoked	Nominal

4	Severity reaction	The clinically graded allergic/adverse reaction	Ordinal

5	Age of the 1st antibiotic use (y)	Age when the first antibiotic was used	Numeric

6	Other allergic disease (skin)	Does a child have some other allergic disease? (manifestation on the skin)	Nominal (Yes, No)

7	Other allergic disease (rhinitis)	Does a child have some other allergic disease? (in the form of allergic rhinitis)	Nominal (Yes, No)

8	Other allergic disease (bronchitis)	Does a child have some other allergic disease? (in the form of obstructive bronchitis)	Nominal (Yes, No)

9	Other allergic disease (asthma)	Does a child have some other allergic disease? (in the form of asthma)	Nominal (Yes, No)

**Table 3:** The attributes used in the dataset (10-17).

10	Blood test on allergy - IgE	Have the antibodies of the IgE type (which usually raise in allergic diseases) been measured?	Nominal (Positive, Negative)
11	Perinatal disorders	Disorders occurring during delivery and the first hours after the birth	Nominal (Yes, No)

12	The child birth order	Born as the first, or the second, etc., child in order	Ordinal

13	Severe respiratory disease	A respiratory disease which is severe enough to be a life frightening (e.g. laryngitis, pneumonia)	Nominal (Yes, No)

14	Age of severe respiratory disease	Age when some type of severe respiratory disease occurred	Numeric

15	Otits media	Otits media	Nominal (Yes, No)

16	Age of otitis media	Age when otitis media occurred	Numeric

17	Other infections	Had there been some other infection before the allergic/adverse reaction on antibiotics occured?	Nominal (Yes, No)

**Table 4:** The attributes used in the dataset (17-26).

18	Other infections (the number of episodes)	How many episodes of infections had there been before the allergic/adverse reaction on antibiotics occurred?	Nominal
19	Varicella	Did the varicella infection occur?	Nominal (Yes, No)

20	Age of varicella	Age when varicella infection occurred	Numeric

21	Hospitalization <2y of age	Hospitalization in the very early childhood	Nominal (Yes, No)

22	Number of infections per year	An average number of infections per year in a particular child, independently on when the allergic/adverse reaction on antibiotics occurred	Numeric

23	Antibiotic exposure before ARA	How many times antibiotics had been prescribed before the allergic/ adverse reaction on antibiotics occurred?	Ordinal

24	Family history on ARA	Family history on allergic/adverse reactions on antibiotics	Nominal (Positive, Negative)

25	Allergic diseases in family	Have there been other allergic diseases in family members?	Nominal (Yes, No)

26	Chr diseases diseases in family	Have there been other chronic diseases in family members?	Nominal (Yes, No)

K-means algorithm needs the number of clusters (*k*) in the data to be pre-specified. Finding the appropriate number of clusters for a given dataset is generally a trial and error process made more difficult by the subjective nature of deciding what 'correct' clustering[32]. The performance of a clustering algorithm may be affected by the chosen value of *k*. Reported studies on k-means clustering and its applications usually do not contain any explanation or justification for selecting particular values for *k *[32].

"The k-means algorithm implementation in many data analysis software packages requires the number of clusters to be defined by the user"[32]. "To find a satisfactory clustering result, usually, a number of iterations are needed where the user executes the algorithm with different values of *k "*[32]. In order to evaluate the performance of simple k-means algorithm in our study, two test modes were used, training set and percentage split (holdout method). The training set refers to a widely used experimental testing procedure where the database is randomly divided into k disjoint blocks of objects, then the data mining algorithm is trained using k-1 blocks and the remaining block is used to test the performance of the algorithm, this process is repeated k times[33]. At the end, the recorded measures are averaged. It is common to choose k = 10 or any other size depending mainly on the size of the original dataset[33].

In percentage split (holdout method), the database is randomly split into two disjoint datasets[33]. The first set, which the data mining system reveals knowledge from the training set. The revealed knowledge may be tested against the second set which is called test set, it is common to randomly split a dataset under the mining task into 2 parts and has 66% of the objects of the original database as a training set and the rest of objects as a test set[33]. Once the tests were carried out using our dataset, results were collected and an overall comparison was conducted [33].

We also tried different number of clusters (2<=*k *< = 5) for each test mode and we observed the results of number of iterations, sum of squared errors and runtime. Sum of squared error (SSE) is an evaluation measure that determines how closely related are objects in a cluster[34].

**Figure 1:**
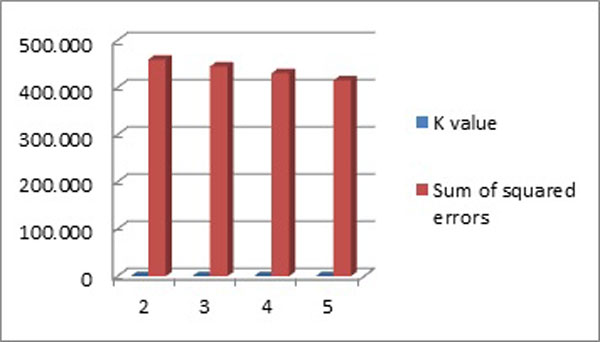
Sum of squared errors and k values with training set mode

The results after analysis are described in Table 5 and 6. We compared the results of the number of clusters obtained by simple k-means algorithm and we found that greater number of clusters produced smaller sum of squared errors. For example, when k value is 2 which is default in Weka, sum of squared error is 459.114, on the other hand, when k value increased to 4, new value of sum of squared error is 430.279(Figure 1). Table 5 shows clusters with training set mode and with k = 4.

**Table 5:** Evaluation of cluster analysis with percentage split test set mode

K value	Number of iterations	Within cluster sum of squared errors	Runtime (Seconds)
2	3 3	459.114(66%) 293.226(34%)	0

3	4 5	444.553(66%) 279.846(34%)	0.01

4	3 5	430.279(66%) 264.258(34%)	0.01

5	5 2	415.160(66%) 248.785(34%)	0.01

The results of the k-means algorithm revealed some patterns in the survey data and four clusters were generated (Table 6). According to the results, some types of antibiotics form their own clusters such as cef&pen, pen, fenoksi and ampicilin. Medical researchers and clinicians can consider and explore these patterns to create some medical ideas.

**Table 6:** Clusters obtained by k-means algorithm with training set mode and k = 4

Attribute	Cluster1	Cluster2	Cluster3	Cluster4
Age	14.5	14.0	16.0	16.0

Age of ARA	5.0	6.0	0.4	<1

Type of antibiotic	cef&pen	pen	fenoksi	ampicillin

Severity reaction	skin	skin	skin	skin

Age of the 1st antibiotic use (y)	5.0	<1	<1	<1

Other allergic disease (skin)	yes	no	no	yes

Other allergic disease (rhinitis)	no	no	no	no

Other allergic disease (bronchitis)	no	no	no	yes

Other allergic disease (asthma)	no	no	no	no

Blood test on allergy - IgE	positive	positive	positive	positive

Perinatal disorders	yes	no	no	yes

Birth order	1	1.379	1.2828	1.2685

Severe respiratory disease	yes	no	no	Yes

Age of severe respiratoy disease	1.5	6.0	6.0	6.0

Otitis media	yes	yes	no	Yes

Age of otitis media	9.0	<1	<1	<1

Other infections	yes	yes	yes	Yes

Other infections	1X	2X	2X	2X

Varicella	yes	no	yes	no

Age of varicella	7.5	3.0	3.0	3.0

Hospitalization <2y of age	no	no	no	no

Number of infections per year	3-4X	2-3X	2-3X	3X

Antibiotic exposure before ARA	2X	1X	1X	1X

Family history on ARA	negative	negative	negative	positive

Allergic diseases in family	no	no	no	no

Chr diseases diseases in family	no	no	no	no

## Evaluation of clustering results

One of the main issues in cluster analysis is the evaluation of clustering results to find the partitioning that best fits the underlying data [35]. "There are three types of validity methods:1) External validity indexes, 2) Internal validity indexes, 3) Relative validity indexes" [36].

External cluster validity metrics use some predefined knowledge, for example, class labels or number of clusters for quality evaluation. In this case, good cluster structure means the same as predefined class structure in the data set. Popular external indexes are Rand index, Jaccard index and Fowlkes-Mallows index. Internal approach evaluates clustering results in terms of quantities that involve the vectors of dataset themselves (e.g. proximity matrix [36].

The main idea of relative approach is the evaluation of cluster structure by comparing it with other cluster structures, resulting by the same algorithms but with different input parameters or by the different algorithms.

In this study, we used external cluster validity methods such as Rand Index, Jaccard Index and F-measure and then compared k-means algorithm results with other clustering algorithms

## Rand index

"This index measures the number of pair wise agreements between the set of discovered clusters K and a set of class labels C, is given by:

$$\text{R = }\frac{a+d}{a+b+c+d}$$

Where *a *denotes the number of pairs of data points with the same label in *C *and assigned to the same cluster in *K, b *denotes the number of pairs with the same label, but in different clusters, *c *denotes the number of pairs in the same cluster, but with different class labels and *d *denotes the number of pairs with a different label in *C *that were assigned to *a *different cluster in *K*"[37,38]. The index results in 0≤R≤1, where *a *value of 1 indicates that *C *and *K *are identical. A high value for this index generally indicates a high level of agreement between a clustering and the natural classes [37,38].

## Jaccard index

"Jaccard index, used to assess the similarity between different partitions of the same dataset, the level of agreement between a set of class labels C and a clustering result K is determined by the number of pairs of points assigned to the same cluster in both partitions:

$$\text{J}=\frac{a}{a+b+c}$$

Where *a *denotes the count of pairs of points with the same label in *C *and assigned to the same cluster in *K, b *denotes the count of pairs with the same label, but in different clusters and *c *denotes the number of pairs in the same cluster, but with different class labels"[37-39]. The index results in 0≤J≤1, where a value of 1 indicates that *C *and *K *are identical [37-39].

## Fowlkes-Mallows index

Let *K *the set of discovered clusters and *C *be the set of class labels. Let *A *be the set of all the data point pairs corresponding to the same class in *C*, and *B *the set of all the data point pairs corresponding to the same cluster in *K*[37]. Then the probability that a pair of vertices which are in the same class under *C*, are also in the same cluster under *K *is given by:

$$\text{P}\left(\text{C},\text{K}\right)=\frac{\left|A\cup B\right)}{\left|A\right|}$$

It is clear that this equation is asymmetric, i.e. *P(C,K) ≠ P(K,C)*, Fowlkes-Mallows Index is defined as the geometric mean of *P(C,K) *and *P(K,C)*:

$$\text{P}\left(\text{C},\text{K}\right)=\sqrt{P\left(C,K\right)*P\left(K,C\right)}$$

The value of the Fowlkes-Mallows Index is between 0 and 1, and a high value means better accuracy [37].

## K-medoids algorithm

k-medoids is a kind of k-means clustering approach and conventional partitioning technique of clustering that clusters the data set of *m *data points into *k *clusters. "It attempts to minimize the squared error, which is the distance between data points within a cluster and a point designated as the center of that cluster"[37]. In contrast to the k-means algorithm, k-medoids algorithm selects data points as cluster centers(or medoids). A medoid is a data point of a cluster, whose average dissimilarity to all the other data points in the cluster is minimal i.e. it is a most centrally located data point in the cluster [37].

## K-median clustering algorithm

K-median clustering algorithm is a type of k-means clustering method like k-medoids algorithm and it calculates the median for each cluster and determines its centroid.

## Single link clustering algorithm

Single link clustering algorithm performs single-link (nearest-neighbour) cluster analysis on an arbitrary dissimilarity coefficient and produces a representation of the resultant dendrogram which can readily be converted into the usual tree-diagram [40].

We conducted the performance evaluation of the following clustering techniques: k-means, k-medoids, k-medians and single link clustering with external cluster validity metrics (Table 7). According to Table 7, k-medians algorithm provides maximum values for all of the external validity metrics and hence outperforms other techniques.

**Table 7:** Evaluation metrics for clustering algorithms

Algorithm	Jaccard Index	Fowlkes-Mallows	Rand Index
K-means	0.5837	0.5350	0.5837

K-medoids	0.3750	0.6124	0.3750

K-medians	0.6033	0.7767	0.6033

Single link clustering	0.0227	0.1508	0.0227

## Discussion

This is a collaborative study of an interdisciplinary team, composed of informaticians and a physician (a GP). The role of a physician was in forming a research question and data collection and in providing comments on health-related issues.

An overall frequency of ARA on antibiotics of 3,15% was observed. Rarely available data for the paediatric population indicates the overall incidence of 9,35% in hospitalized children and 1,46% in outpatients [33]. Many factors can affect the variation in the frequency of this disorder, including the children age (as shown in our paper), the natural distribution of risk factors in the population, the types of antibiotics prescribed, the custom of ARA recording and physicians' education on both, symptoms and mechanisms of ARA and antibiotic prescription [34].

When all four clusters in parallel were put into consideration, some general rules, in regard to ARA in children, could be observed. As the first, there were two time peaks of the ARA occurrence: in the year of birth and in the late pre-school age (around 5-6 y). In older children with ARA, the causing antibiotics were classified as with higher historical use (penicillin).

Some common characteristics of children with ARA might include: 1) predisposition to allergic disorders (positive IgE blood test), 2) however, not manifested with allergic respiratory diseases (hay fever and asthma). This connection should be taken into account even if it is known that allergic diseases show the time-dependent occurrence during the childhood (the so-called "allergic march", manifested as a progression of atopic diseases from eczema to asthma), for reason that the current age of cases with ARA corresponds with adolescence (14-16 year). These results seem contrary to what is known from the early studies, that atopic subjects do not show higher incidence of penicillin allergy, in comparison to the general population [6]. It cannot, in fact, be known, from our results, whether atopy in children can also increase their predisposition for ARA on drugs (especially on antibiotics other than penicillin), or whether, on the contrary, early antibiotic exposure increases the risk for atopic diseases, as postulated traditionally [19]. Or, these results may be only due to the confounding effects, consequently to the predominant use of -lactam antibiotics in children. Namely, undesirable reactions on these antibiotics are known as being predominantly caused by allergic mechanisms, usually mediated with IgE antibodies [6]. Nevertheless, these results can direct future prevention strategies, mainly by means of preserved prescriptions of antibiotics in children with increased IgE antibodies.

Other constant and common features of children with ARA include: 3) at least one episode of infections (other than respiratory infections, also including otitis media) experienced before the time of ARA occurrence, as well as an early antibiotic use (in the first year of life). These results might be reflective of the immune system disturbation, in the early childhood, which can increase the chance for both, ARA on antibiotics and infections. Also, there are information that some infections can serve as a promoting factor, by ensuring conditions for the immune reaction on a drug to start, which otherwise could not be the case [5]. In addition to these explanations, the second result might also implicate the increased risk for ARA to occur, through the negative effect of an early antibiotic use on the commensal intestinal flora and the subsequent impairments of the immune system development [19], [20].

Some additional factors, found to commonly occur in children with ARA, include: 4) frequent infections (defined as two or more times per year), reflecting poor hygiene, or the immune system dysfunction, and 5) low antibiotic pre-exposure counts (1-2 times), indicating sensitizing reaction as the possible mechanism of ARA. In accordance to the latter, it is commonly known that patients usually develop allergic reactions when reexposed to an antibiotic [6].

When clusters 3 and 4, representing an early onset of ARA (during the first year of life), were compared to each other, somewhat different patterns were obtained, probably indicating different mechanisms between ARA on ampicillin (a broad-spectrum antibiotic) and fenoksimetilpenicilin (a narrow-spectrum penicillin for an oral use). Otherwise, these antibiotics share the common structure, that of the -lactam antibiotic group, also sharing some common features [5].

In regard to ampicillin, other allergic diseases, including skin eczema and obstructive bronchitis (both disorders occurring early along the course of the "allergic march"), may contribute to the onset of ARA. These results are likely to support the hypothesis, already presented above, about the common pathogenetic background of both, atopic diseases and ARA on antibiotics, in children. As an alternative explanation of this connection, evidence has been provided by many clinical studies, although not consistently, that antibiotic exposure in early infancy is likely to increase the risk for childhood atopy [19], [23]. This inconsistency in knowledge gained on this issue, might be the consequence of the different behavior of otherwise similar substances, such as in our study the case with fenoksimetil penicillin (cluster 3) and ampicillin (cluster 4). The unfavorable drug reaction, in ampicillin risk group (cluster 4), according to our results, might also be supported with the existence of perinatal disorders, implicating immunodeficiency and obstacles in the postnatal immune system development. In numerous studies, conducted to-date, an attention has not been paid to the importance of these very early developmental disturbances. Furthermore, our results also indicate that the occurrence of otitis media in early life (in some reports considered as the complication of influenza virus infection and, as such, the manifestation of the immune system dysfunction) can also be considered as a contributing factor for the early onset of ARA on ampicillin (cluster 4). This risk group, in contrast to the comparative one, for the time of onset (cluster 3), was also prone to the development of severe respiratory disease, although with the onset later in life (at six age), further indicating immunodeficient disorders. When positive family history on ARA is added to this risk group (cluster 4), this all together indicates that a set of inherited and acquired immune system disorders can be important for the occurrence of ARA on this broad-spectrum antibiotic.

Some elements of this pattern, associated with ARA on ampicillin (cluster 4), can be recognized as a part of the cluster describing ARA on cefalosporins (cef&pen, cluster 1), another broad-spectrum group of antibiotics. These elements, overlapping between the two clusters, include perinatal disorders and severe respiratory disease, although here, the severe respiratory disease preceded (and probably contributed to) the onset of ARA (cluster 1). The combined cef&pen ARA event probably means allergic cross-reaction that may occur between penicillin and cephalosporins of the older generation [6].

Also, it is interesting to observe that two very similar antibiotics, from the common penicillin groups (clusters 2 and 3), have gained much of the similarity in their risk factors patterns.

These results, indicating multiple factors clustered within distinct patterns, each of them specifically associated with a particular risk group (or an antibiotic), are similar to the results of the studies on the association of an early antibiotics use and the occurrence of allergic diseases later in the childhood. According to these studies, a complex cause/outcome model should be formed, in order to make conclusions on this issue, and it is not possible to achieve by analyzing only one, or even a few risk factors [19], [20], [24].

All these factors, extracted from the health records and selected within four clusters, reflect patients' (children's) clinical and pathophysiological features. We can speculate that the reason why ARA on some other antibiotics, also listed above, have not been presented with a cluster, might be the need for different clinical parameters selection, those ones not recorded in the health records. Alternatively, some other factors could be responsible for ARA, such as, for example, differences in pharmacodynamic mechanisms of drug action. In contribution to this latter explanation, very low ARA rates for macrolide antibiotics have been reported [5].

Results of this study have confirmed some relatively known facts about ARA in children, including the influence of early life infections and antibiotic prescriptions, as well as the predomination of allergic mechanisms underlying ARA, mostly mediated with IgE antibodies. The nature of the association between atopy and ARA in children, also important for understanding childhood allergic diseases, remain to be elucidated in the future. In fact, our results indicate that this association might be important only for early ARA onset (in the first year of life) and for a particular antibiotic used. The main contribution of this paper is in the results clearly showing for the first time that only a cluster of factors can explain ARA, specifically for a particular children group, or an antibiotic.

Results of this study can further be utilized for planning future research on this issue. They can also be useful when preparing recommendations for antibiotics prescription and to guide the standardized health data record. Merely an increase in awareness of physicians on risk factors for ARA in children can be sufficient to change their attitudes towards antibiotics prescription. Computer-based tools would be helpful in many aspects when managing these issues, especially by means of the possibility for systematic data recording and data modeling, suitable for the purpose of prediction and risk factors identification. Also important would be the drug allergy alert and prescription support systems, as well as programs for education promotion [41], [42].

We analyze health records created in a health center in East Croatia to explore new knowledge for adverse reactions and allergy (ARA) on antibiotics in children. The broad application of business enterprise hospital information systems utilizes large amounts of medical documents, which need to be reviewed, observed, and analyzed by human experts. There is need for some techniques which provide the quality-based discovery, the extraction, the integration and the use of hidden knowledge in those documents [43]. Human-Computer Interaction and Knowledge Discovery along with Biomedical Informatics are of increasing importance to effectively gain knowledge, to make sense out of the big data. In the future, we can combine these fields to support the expert end users in learning to interactively analyze information properties thus enabling them to visualize the adverse reactions and allergy (ARA) on antibiotics data [44].

## Conclusions

Biomedical research aims to search new and meaningful knowledge to provide better healthcare [45-47]. Adverse reactions and allergy (ARA) from antibiotics in children is an important research issue for the medical domain. In this study, we targeted on knowledge discovery for this problem and perform a study based on data mining to predict clusters in the survey data extracted from health records of children in Eastern Croatia.

We used computational techniques and then applied k-means algorithm to the dataset to generate some clusters which have similar features. Our results highlight that some type of antibiotics form different clusters. Medical researchers and pharmaceutical companies can utilize and interpret our results. Despite that our study has some limitations, for example we have small dataset consisting of 42 instances, we hope that we can extend the dataset and apply data mining algorithms on it in the future.

In conclusion, we believe that our study can be good example on data mining for adverse reactions and allergy (ARA) from antibiotics in children.

## Competing interests

The authors declare that they have no competing interests.

## Authors' contributions

Authors contributed equally to this work.

## Declarations

Publication for this article has been funded by the hci4all.at Group

This article has been published as part of *BMC Bioinformatics *Volume 15 Supplement 6, 2014: Knowledge Discovery and Interactive Data Mining in Bioinformatics. The full contents of the supplement are available online at http://www.biomedcentral.com/bmcbioinformatics/supplements/15/S6.
